# Requirements and ontology for a G protein-coupled receptor oligomerization knowledge base

**DOI:** 10.1186/1471-2105-8-177

**Published:** 2007-05-30

**Authors:** Lucy Skrabanek, Marta Murcia, Michel Bouvier, Lakshmi Devi, Susan R George, Martin J Lohse, Graeme Milligan, Richard Neubig, Krzysztof Palczewski, Marc Parmentier, Jean-Philippe Pin, Gerrit Vriend, Jonathan A Javitch, Fabien Campagne, Marta Filizola

**Affiliations:** 1 Department of Physiology & Biophysics, Weill Medical College of Cornell University, New York, NY, USA; 2 HRH Prince Alwaleed Bin Talal Bin Abdulaziz Alsaud Institute for Computational Biomedicine, Weill Medical College of Cornell University, New York, NY, USA; 3 Department of Biochemistry and Groupe de Recherche Universitaire sur le Médicament, Institute for Research in Immunology and Cancer, Université de Montréal, Montréal, Quebec, Canada; 4 Department of Pharmacology and Biological Chemistry, Mount Sinai School of Medicine, New York, NY, USA; 5 Department of Pharmacology, University of Toronto, Toronto, ON, Canada; 6 Institute of Pharmacology and Toxicology, University of Wurzburg, Wurzburg, Germany; 7 Molecular Pharmacology Group, Division of Biochemistry and Molecular Biology, Institute of Biomedical and Life Sciences, University of Glasgow, Glasgow, Scotland, UK; 8 Department of Pharmacology, University of Michigan, Ann Arbor, MI, USA; 9 Department of Pharmacology, School of Medicine, Case Western Reserve University, Cleveland, Ohio, USA; 10 Institut de Recherche Interdisciplinaire en Biologie Humaine et Moléculaire (IRIBHM), Université Libre de Bruxelles, Brussels, Belgium; 11 CNRS Unité Mixte de Recherche 5203, INSERM U661, Universités de Montpellier 1 et 2, Montpellier, France; 12 Center for Molecular and Biomolecular Informatics, Radboud University Nijmegen, Nijmegen, The Netherlands; 13 Center for Molecular Recognition, Columbia University College of Physicians and Surgeons, New York, NY, USA

## Abstract

**Background:**

G Protein-Coupled Receptors (GPCRs) are a large and diverse family of membrane proteins whose members participate in the regulation of most cellular and physiological processes and therefore represent key pharmacological targets. Although several bioinformatics resources support research on GPCRs, most of these have been designed based on the traditional assumption that monomeric GPCRs constitute the functional receptor unit. The increase in the frequency and number of reports about GPCR dimerization/oligomerization and the implication of oligomerization in receptor function makes necessary the ability to store and access information about GPCR dimers/oligomers electronically.

**Results:**

We present here the requirements and ontology (the information scheme to describe oligomers and associated concepts and their relationships) for an information system that can manage the elements of information needed to describe comprehensively the phenomena of both homo- and hetero-oligomerization of GPCRs. The comprehensive information management scheme that we plan to use for the development of an intuitive and user-friendly GPCR-Oligomerization Knowledge Base (GPCR-OKB) is the result of a community dialog involving experimental and computational colleagues working on GPCRs.

**Conclusion:**

Our long term goal is to disseminate to the scientific community organized, curated, and detailed information about GPCR dimerization/oligomerization and its related structural context. This information will be reported as close to the data as possible so the user can make his own judgment on the conclusions drawn for a particular study. The requirements and ontology described here will facilitate the development of future information systems for GPCR oligomers that contain both computational and experimental information about GPCR oligomerization. This information is freely accessible at .

## Background

**Figure 1:**
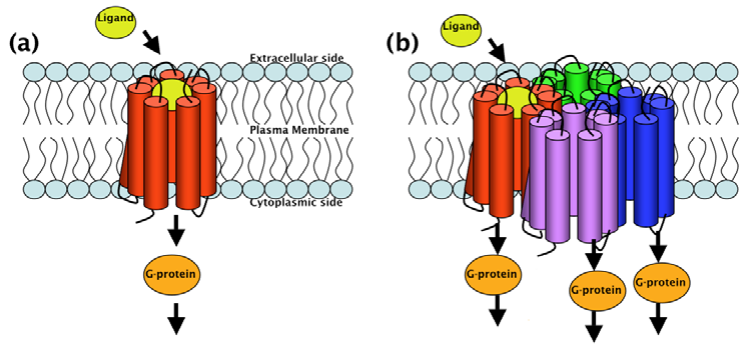
**Traditional and current views of GPCR signaling**. The traditional view of GPCR signaling assumed that monomeric receptors participated in ligand binding and signal transduction. The current view suggests that GPCRs may form homo- and/or hetero-oligomers, and that ligand(s) binding to one or more receptors may activate neighboring receptors in the oligomeric complex.

G Protein-Coupled Receptors (GPCRs) are among the largest and most diverse protein families in mammalian genomes, and constitute the largest single family of therapeutic targets for drug treatment. They are integral membrane proteins with a central common core made of seven transmembrane helices connected by intracellular and extracellular loops (Figure 1). The primary function of GPCRs is to transduce extracellular stimuli into intracellular signals through G-protein dependent and independent pathways.

The traditional view of GPCR function had focused on the assumption that monomeric receptors participated in ligand binding and signal transduction processes (Figure 1a). Specifically, a single ligand was assumed to activate a single receptor by producing a conformational change in the receptor that would induce activation of a G protein or effector. Although it is well accepted that a monomeric GPCR can activate heterotrimeric G-proteins, the view that they function only as monomers in cells has recently been challenged by the discovery that numerous GPCRs form homo- and/or hetero-oligomers (Figure 1b). Functional crosstalk between protomers in a dimeric or oligomeric complex has been described, and it is possible that ligand binding to one or more receptors may activate neighboring receptors in an oligomeric complex, giving rise to a cascade of interconnected signaling events.

The physiological relevance of GPCR oligomerization is largely accepted nowadays, especially in light of the discovered functional implications of GPCR association that include pharmacological diversity, G-protein coupling specificity, downstream signaling amplification or attenuation, and/or internalization (see [1-9] for recent reviews). This growing amount of information and its required incorporation into physiologically relevant functional models of GPCRs encourages the development of a publicly accessible repository of information focused on GPCR homo- and hetero-oligomers.

Currently available bioinformatics tools for data storage devoted to GPCRs have been designed with a strong emphasis on receptor monomers. For example, information repositories such as GPCRDB [10] and tGRAP [11] include sequence data, alignments of monomers, 2D visualization of monomer units, and mutation information for receptor mutants. The NC-IUPHAR (The International Pharmacology Committee on Receptor Nomenclature and Classification) database on GPCR nomenclature and drug classification [12] includes the most commonly studied GPCRs with a particular focus on information about their pharmacological and functional properties, and their function, and localization *in vivo*. These information systems have proven extremely useful to support structural and functional studies of GPCRs, as shown by their strong user base. For instance, GPCRDB has established itself as a central repository for GPCR information with peak access at some 100,000 requests per month [10]. Information systems such as GPCRDB provide users with a unified view of information in a given field (or for a class of molecules), and constitute frequently updated resources that support structured queries and provide advanced visualizations.

Given that physiologically relevant functional models of GPCRs must incorporate GPCR homo- and/or hetero-oligomeric constructs, the explosion of information about GPCR oligomerization makes this an opportune time for the development of a web-based information system that specifically a) stores computational and experimental information about GPCR oligomerization, and b) allows browsing, visualization and structured querying of its contents. Such a system would foster and support productive and quantitative communication and collaboration between computational and experimental scientists, but is not currently available in any form. To best serve the broad GPCR community by providing a comprehensive and reliable (data-driven, rather than interpretation-driven) GPCR oligomerization information system, it is important that opinions from GPCR experts in both experimental and computational fields are given due consideration. This manuscript describes the result of a community dialog we initiated to identify the requirements for a comprehensive GPCR oligomerization information system and to design an ontology (information scheme) to represent such information. The GPCR oligomerization ontology that we present here is a formal description of the information concepts required to describe GPCR oligomerization and of the relationships between these concepts. As such, this ontology allows the organization of information in a formal way, a prerequisite to supporting many of the requirements that potential users identified for the future GPCR oligomerization information system (see Information System Requirements below).

## Results

An information system devoted to homo- and hetero-oligomers of GPCRs requires the capability to store, and allow browsing, visualization and structured querying of a specialized set of heterogeneous data. To facilitate the implementation of a comprehensive information system focused on GPCR oligomerization, it is important to make informed decisions about the specific usages the information system will support and what type of information must be stored and queried. Thus, in a first step towards developing such a system, we have obtained system requirements – a list of features essential to the finished information system – from experts in the GPCR oligomerization field, and have developed an ontology to represent the results of GPCR oligomerization studies (both experimental and computational). The system requirements and ontology constitute a comprehensive information management scheme for a GPCR oligomerization information system and can be used to guide the fine design and implementation of such a system. Throughout the ontology and this manuscript we define the word 'protomer' to refer to a protein which is a constituent part of an oligomer. In contrast, we reserve the word 'monomer' for stand-alone GPCR proteins.

### Information system requirements

In the following sections, we report the system requirements that have been identified for a comprehensive GPCR oligomerization information system. Each requirement describes a high-level feature that an information system for GPCR oligomers must include to be useful, and explains how this feature will support studies of GPCR oligomers.

#### 1. Providing an electronic repository of both experimental and computational information about GPCR oligomers

The repository will complement the scientific literature by offering a unified view of the data and/or conclusions published in different articles. One of the most daunting obstacles encountered when directly searching the literature is the existence of varying monomer/oligomer naming schemes or different residue numbering conventions. This makes it extremely difficult to locate articles of interest using current text searching methods. A useful GPCR oligomerization information system should make it possible to bypass this problem by supporting the creation of unified views by aggregating data along different axes. In an information system for GPCR oligomers, aggregation could be done at the level of the oligomer or structural domain, so that obtaining the list of small molecules that were shown to bind an oligomer would be possible even if the information was originally published in five articles where the same oligomer was referred to by three different names. The GPCR oligomerization information system should therefore eliminate the common problems encountered when searching the literature with current text searching methods. A GPCR information system should also provide links to the primary literature for each fact or interpretation offered.

Native oligomeric complexes of GPCRs that satisfy any or all of the rules recently stipulated by NC-IUPHAR [13] should and will be properly highlighted.

#### 2. Linking and complementing existing GPCR resources that provide information about GPCR monomers

There is a rich compendium of established and experimental bioinformatics resources designed to support GPCR research (e.g., GPCRDB [14], tGRAP [11], GPCRIPDB [15], NC-IUPHAR [12], Arcadia [16]). A GPCR oligomerization information system should not duplicate existing monomer resources, but rather should focus on interfacing with these resources to import data or to link to them to relate oligomer-specific data to known facts about their constituent protomers. This information is needed to reveal possible changes in the structure-function relations of a specific GPCR oligomer compared to its monomeric forms. Information available about the monomers found in existing resources includes but is not limited to: transmembrane (TM) helical boundaries, sequence alignments, phylogenetic trees, predictions of solvent accessibility of TM residues, snake-like diagrams, three-dimensional (3D) molecular models, Protein Data Bank (PDB) files associated with GPCRs, point mutations, GPCR interacting partners, GPCR cDNAs, chromosomal locations, and ligand-binding constants. Similarly, a GPCR oligomerization information system should provide the capability for others to define links to data entries in the information system. For instance, in the systems to which GPCRDB links, this requirement is fulfilled when linked resources export a catalog of the internal and external accession codes for each data item that they contain. Developments at GPCRDB, such as the creation of web services, will facilitate this integration with GPCR-OKB.

#### 3. Providing information about the experimental details of the method used in published studies of GPCR oligomers

A variety of experimental procedures and systems has been used to study GPCR oligomers. A comprehensive information system involving GPCR oligomers should include information about: i) the experimental procedure used to characterize the oligomer, e.g., Fluorescence Resonance Energy Transfer (FRET), Bioluminescence Resonance Energy Transfer (BRET), Time Resolved FRET (TR-FRET), cross-linking, co-immunoprecipitation, co-expression of fragments or modified protomers, Atomic Force Microscopy (AFM), or use of dimer-specific antibodies (for a complete review see [17]); ii) the biological system studied, e.g., native tissue/transfected cells; and iii) the evidence available for cross-modulation of ligand binding, activation, internalization, e.g., see [18,19]. The user should be able to search for single or multiple experimental details, in order to identify the multimeric complexes that have been studied under specified conditions. Knowledge of experimental details used in published studies of GPCR oligomers will allow users to have a better understanding of the corresponding results. In addition, it will assist the user in the design of directed pharmacological and physiological experiments for the specific multimeric system under study.

#### 4. Providing experimental and computational results rather than interpretation of those data

Scientific publications contain descriptions of experimental results and of the methods used to produce them. In contrast, it is not unusual for the interpretation of the data presented in a manuscript to be challenged in follow-up publications, as more experimental data become available. Because various interpretations of the data are often the object of debate until a consensus is reached, the information system will be most helpful if it stores the experimental data. In the case of conflicting data, all experimental results will be reported, with the appropriate reference.

#### 5. Providing information about the specific residues at oligomeric interfaces established or predicted by experimental and/or computational approaches

A detailed information system about GPCR oligomers should include information about the experimental method (e.g., cross-linking [1] or site-directed mutagenesis) or computational procedure (e.g., evolutionary trace (ET) method, correlated mutation analysis (CMA), subtractive correlated mutation (SCM), etc.) used to determine/predict dimerization/oligomerization interfaces [20]. Availability of information about the composition of the interfaces of dimerization/oligomerization will facilitate an understanding of the nature of the interaction between GPCR subunits. This information is essential to the design of oligomerization-disrupting mutants directed towards modulating GPCR function. Interface residues should be identified both by the absolute sequence numbering and by a GPCR generic numbering system, which makes it possible to refer, comparatively, to structurally cognate GPCRs. For family A GPCRs, this generic numbering system consists of assigning each helix residue a number relative to that of the most conserved residue in each transmembrane helix, which is arbitrarily assigned the number 50 [21]. Different sets of index residues are selected for other GPCR families, i.e., family C GPCRs [22]. As generic numbering systems for other families are proposed, they will be incorporated.

#### 6. Providing structural information about physiological GPCR oligomers

Information about the particular experimental method used to obtain the structural information (e.g., X-ray diffraction in the case of the extracellular domain of members of family C GPCRs) and/or the specific type of modeling procedure (e.g., distance-based modeling in the case of rhodopsin) should be stored and detailed as completely as possible. Results from several computational techniques, including molecular dynamics simulations [23], should also be considered for storage as they are expected to help rationalize possible dynamic mechanisms of GPCR oligomers and suggest potential ways of modifying receptor function.

#### 7. Providing information about potential mechanisms of activation of GPCR oligomers

The user should receive detailed information when available about i) activated protomer(s) within the oligomer; ii) the activating ligands; iii) single or multiple occupancy of binding sites; iv) types of conformational change within each protomer; v) symmetric/asymmetric functioning; vi) *cis*- or *trans*-activation; vii) possible structural rearrangement at the interface upon activation; viii) GPCR-G-protein stoichiometry. Although most of this information derives from paradigms of G protein-dependent signaling, inferences from G-protein independent pathways will also be incorporated as more detailed information becomes available.

#### 8. Providing detailed information about functional roles of GPCR oligomers

The user will find beneficial the following information: i) role of oligomerization in maturation and cell-surface delivery; ii) ligand regulation (and characterization of the ligand as agonist or antagonist); iii) cross-modulation of signaling/binding; iv) negative/positive cooperativity; v) attenuation/potentiation of signaling in G protein-dependent and independent pathways; vi) G-protein specificity; vii) internalization (for a recent review see [2]). By searching for single or multiple functional details, the user should easily identify the multimeric complexes that satisfy such criteria.

#### 9. Linking to novel compounds that are proposed to selectively target GPCR oligomers

Recent evidence for compounds that can selectively activate hetero-oligomers but not homo-oligomers suggests that GPCR hetero-oligomers can be used as models in the development of new therapies [24], as well as in the design of new drugs with reduced side effects [8,25,26]. Therefore, the user will find helpful a connection between the detailed information available for these multimeric systems and the compounds they are purported to recognize and bind. To this end, the information system should link to databases (e.g., PubChem [27]) that contain information about the chemical and structural features of both synthetic and natural compounds, as well as their vendor identifier if available. Antibodies that selectively target GPCR oligomers will also be included in the information system.

#### 10. Providing information about the physiological relevance of GPCR oligomers

One of the main requirements for the recognition and acceptance of GPCRs multimeric receptors recently defined by NC-IUPHAR [13] is a firm demonstration of the actual physiological relevance of those oligomeric complexes/receptors. This is especially important in the case of hetero-oligomers, for which most of the results come from heterologous cellular systems where two different GPCRs are co-expressed simultaneously. Therefore, information concerning co-localization studies in native tissues, *in vivo *effects of hetero-oligomer specific ligands as well as reported *in vivo *phenotypical changes (e.g., pharmacological response or cooperativity) in knock-out animals should be available to the user.

### A comprehensive ontology for the study of GPCR oligomers

**Figure 2:**
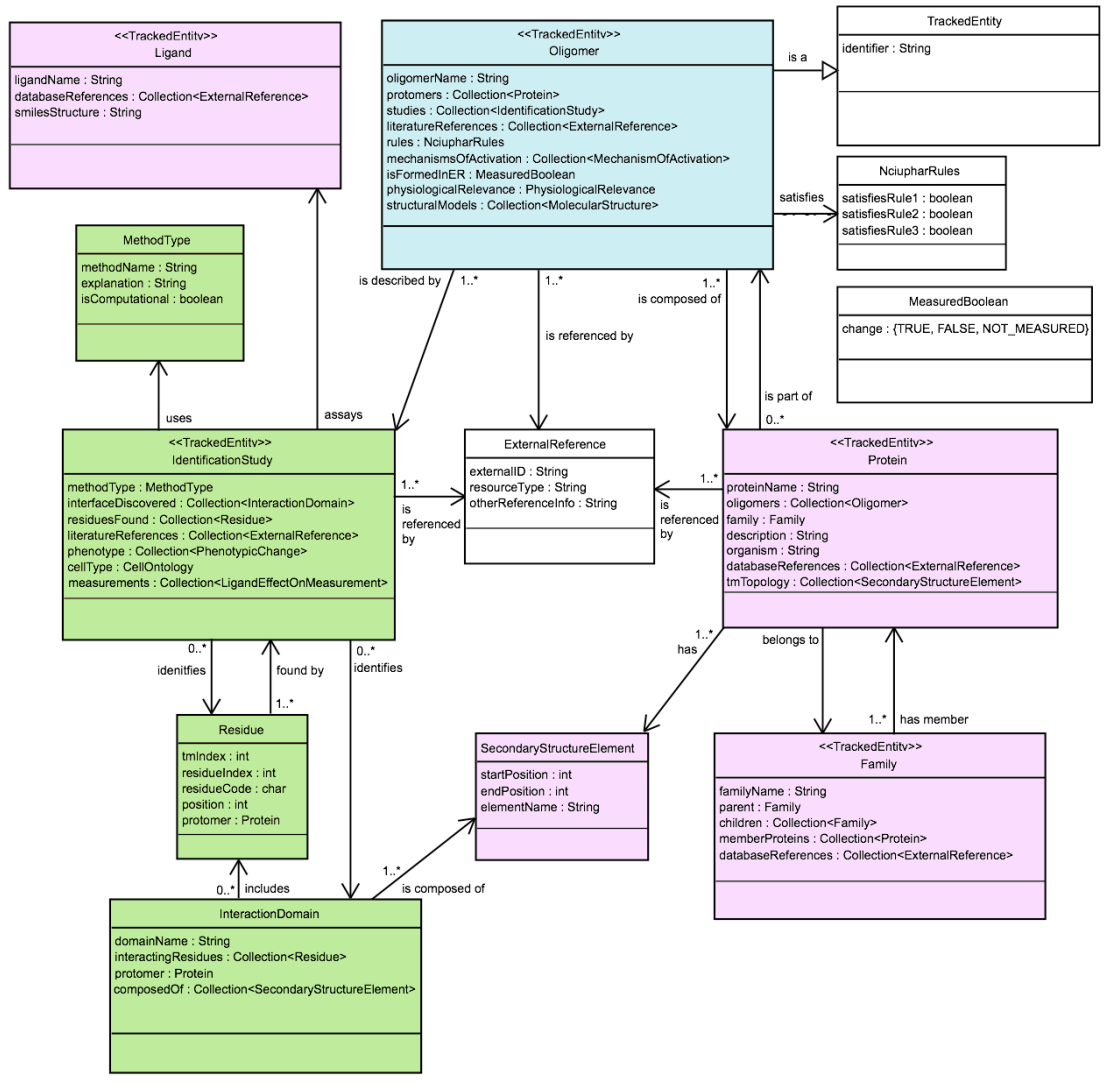
**UML diagram of the Oligomer concept and related concepts**. Each concept is shown as a box, and is named in the top section of the box. All attributes for each concept are listed in the middle section of the box. Arrows represent relationships between concepts, and open-ended arrows indicate "is a" relationships. The relationship of one concept to another is indicated by the text on each arrow. Arrows with [0..*] (zero or more) or [1..*] (one or more) indicate the number of instances of the concept at the end of the arrow that is associated with the concept at the beginning of the arrow. The Oligomer concept is central to the GPCR oligomerization ontology and all other concepts in the ontology relate to it either directly (e.g., Oligomer ''is described by'' IdentificationStudy) or indirectly (e.g., Oligomer ''is composed of'' one or more [1..*] Protein that ''belongs to'' Family).

**Figure 3:**
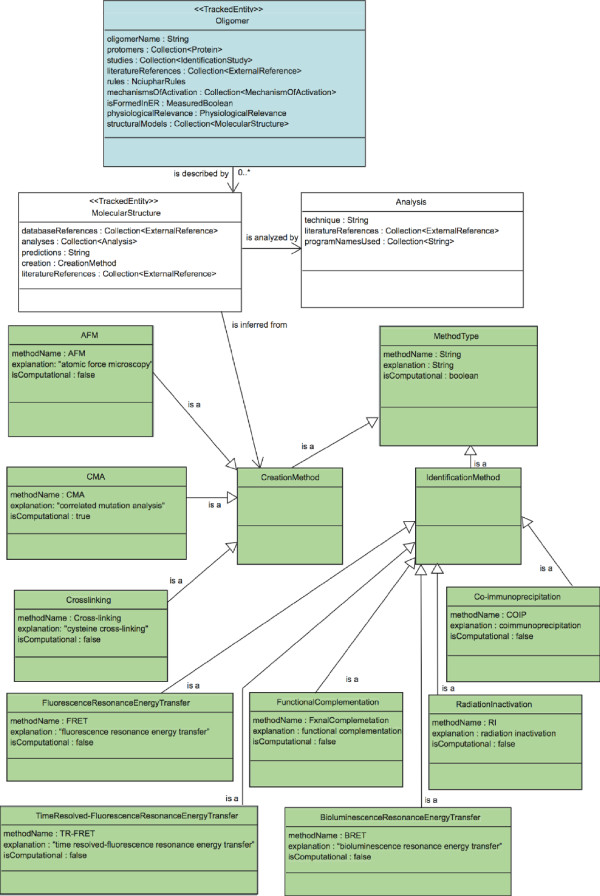
**UML diagram of modeling-related concepts**. Structural models of Oligomers are represented in the GPCR oligomerization ontology by a MolecularStructure concept. Each MolecularStructure is created with an instance of MethodType, and may be analyzed by many computational methods (instances of Analysis). MethodType has two subclasses: IdentificationMethod, which is used to identify the oligomer, and CreationMethod, which is used to create the MolecularStructure. IdentificationMethod and CreationMethod can have many sub-concepts that describe the precise type of method. In this figure we show only a few examples of such concepts.

**Figure 4:**
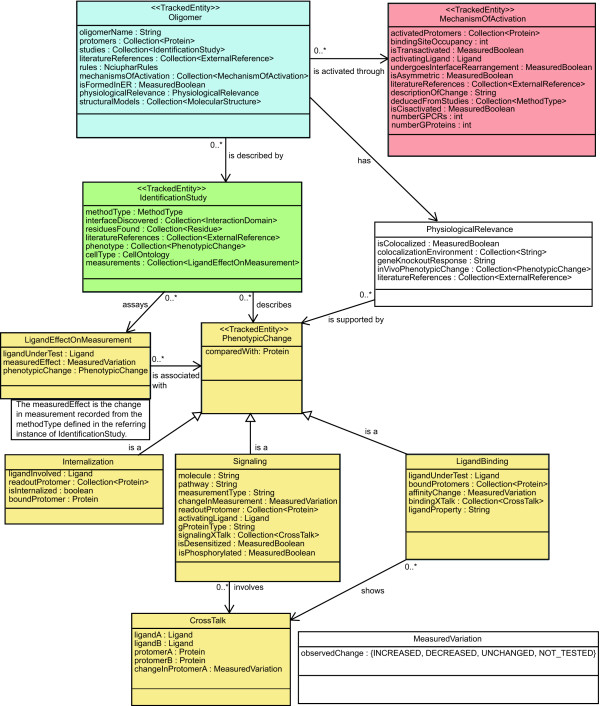
**UML diagram of concepts related to oligomerization-induced phenotype changes**. The GPCR oligomerization ontology focuses on changes in the phenotype that occur when GPCR protomers oligomerize. There are three types of phenotypic change that are described by the ontology: changes in internalization, changes in signaling, and differences in the ligand binding of the oligomer as compared to any of the constituent protomers. The effect that ligand(s) binding to one or more of the protomers in an oligomer may have on binding of ligands to other protomers, or on the change in signaling, is described by the CrossTalk concept. The Internalization concept is used to describe changes that different ligands have on the trafficking of the Oligomer to the cell membrane. Any information that is available about the mechanism of activation of the Oligomer is stored in the MechanismOfActivation concept. The PhysiologicalRelevance concept stores information about the Oligomer *in vivo*.

**Figure 5:**
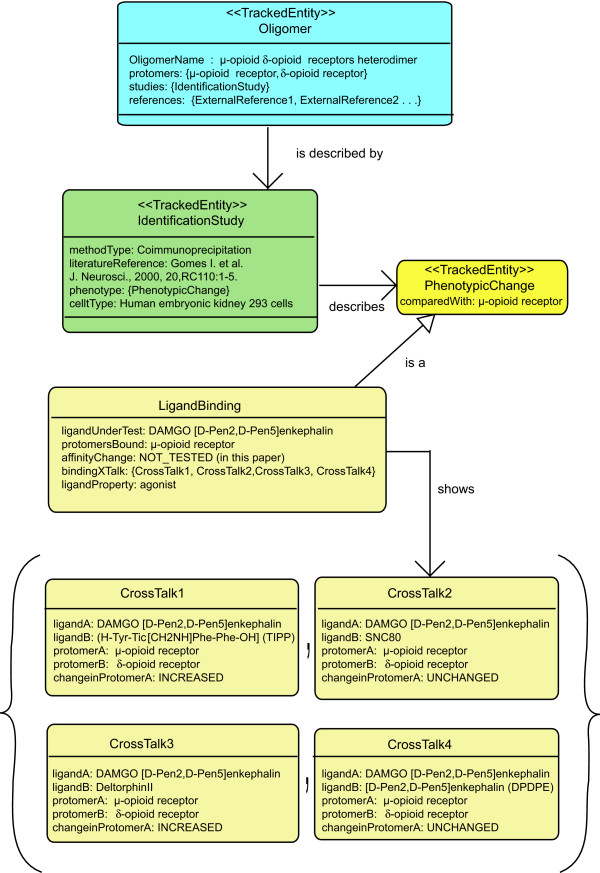
**UML diagram of the oligomerization-induced cooperativity effects in HEK-293 cells co-expressing μ-opioid and δ-opioid receptors**. The attribute *bindingXTalk *compiles any information related to changes in the affinity of the *ligandUnderTest *(DAMGO) in the presence of another ligand (e.g., TIPP Ψ, deltorphin II, SNC80, or DPDPE). Treatment of HEK-293 cells with the selective δ-opioid ligand TIPP Ψ results in increased binding of the μ-opioid agonist DAMGO (see CrossTalk1). Among the δ-opioid selective agonists SNC 80, DPDPE or deltorphin II, only the last leads to a significant increase in DAMGO's binding affinity (see CrossTalk2-4).

The requirements presented in the previous section call for the management of various types of information about the oligomers, their structural domains, or their binding ligands. Because each of these information types relates to one or more other information types, and because the relationship between such types can become complex, it is necessary to specify precisely the information type relationships that the information system will support. To achieve this, we have created a formal ontology for a comprehensive GPCR oligomerization information system currently under development in our laboratories. The term ontology is commonly used in bioinformatics to denote various types of ways to organize biological information (from the directed acyclic graph of the "Gene Ontology" to frame-based systems). Here, we consider that an ontology is the result of making choices to represent a specific aspect of reality, e.g., a biological entity such as a GPCR oligomer within an information system, database, or knowledge representation system, in order to make it amenable to certain types of computations (we therefore follow the definition of an ontology given in [28]). Choices are necessary to simplify the representation so that it only maintains the attributes needed for the type of computational use planned. From the use case scenarios and system requirements obtained as a result of an interdisciplinary effort, we have incorporated the elements of information that are required to describe sufficiently the phenomena of GPCR homo- and hetero-oligomerization, and also how these elements of information combine to form a system that will serve the targeted users. The ontology is a crucial component of the information management scheme for an information system because it formally specifies what can be stored in such a system and what types of computations can be performed with the stored material. The rest of this section describes in detail the ontology developed for a comprehensive GPCR oligomerization information system. The full ontology is depicted in Figures 2, 3, and 4, and is available in OWL format from the project web site [29]. A specific example is illustrated in Figure 5.

#### 1. Ontology primer

##### 1.1 The Oligomer concept

The Oligomer concept is central to the GPCR-OKB, and we use it here to define the conventions followed in the rest of this manuscript. Figure 2 shows the Oligomer concept with its connections to other concepts of the ontology. In this manuscript, all graphical representations of the ontology follow the UML conventions [30]. Briefly, concepts are depicted by rectangular boxes with their corresponding names reported in the top portion of the box. Open-ended arrows from one concept to another indicate that the destination concept is more general than the source concept (those arrows can be read: 'source "is a" destination'). Other arrows indicate relationships between concepts. Attributes of a concept are reported within each concept box. Attributes conventionally begin with lowercase letters. If the attribute name is made up of two or more words, the subsequent words are capitalized to make it easier to understand the name of the attribute. For example, Figure 2 shows that the Oligomer concept has an attribute *oligomerName *of type String that stores the name of the Oligomer. The Oligomer concept also has a *protomers *attribute that lists the specific protein types that form the oligomer. The ontology does not track the exact stoichiometric subunit composition of oligomers because most of the time this information is not known. Instead the ontology records what types of GPCRs are known or predicted to participate in that instance of Oligomer. For instance, to indicate that the δ-opioid receptor is predicted to oligomerize with the μ-opioid receptor, an instance of the Oligomer concept is created with a *protomers *attribute that references these two GPCRs. The *protomers *attribute is of type Collection<Protein>, a notation that indicates that this attribute can contain a number of instances of the Protein concept. The number of Protein instances that can participate in the relation defined by the *protomers *attribute is specified on the arrow from Oligomer to Protein. This is an example of an association between one concept and another, where one concept can have multiple instances of the other concept. In a UML diagram, such multiplicities of association are indicated at the start of the arrow between the two concepts. In this case, the notation 1..* indicates that an instance of Oligomer may have one or more Protein instances in its attribute *protomers*, where the 1 indicates that an Oligomer must have at least **one **Protein associated with it, and the * indicates that there can be **more than one **Proteins associated with the Oligomer. Similarly, it is possible (for example in the association from Protein to Oligomer), that a Protein may not be a part of any Oligomer at all, or may be a component of many different Oligomers. In this case, the notation reads 0..* (see Figure 2).

##### 1.2 ExternalReference

Similarly to the *protomers *attribute, the *literatureReferences *attribute indicates that each instance of the Oligomer concept is associated with one or more (1..*) ExternalReference instances. The *literatureReferences *attribute allows citations and other outside references to be attached to a particular Oligomer instance. The ExternalReference concept performs this function because its three attributes (*externalID*, *resourceType *and *otherReferenceInfo*) allow the identification of published material and external database links associated with that Oligomer instance. For example, an ExternalReference instance whose attributes are {*externalID *= 10926528, *resourceType *= PubMed and *otherReferenceInfo *= ""} refers to an article in PubMed whose primary identifier is 10926528. This information is sufficient to identify the article describing the crystal structure of rhodopsin [31]. Articles that are not indexed in PubMed can also be referenced. A reference to this same article can also be constructed by using a DOI resource type: {*externalID *= 10.1126/science.289.5480.739, *resourceType *= DOI and *otherReferenceInfo *= ""} [32]. The ExternalReference concept thus allows the cross-linking to any piece of information that is available online and is in the public domain. Other types of references that are not accessible through the above mechanism can be cited using the *otherReferenceInfo *attribute.

##### 1.3 TrackedEntity

The TrackedEntity concept shown in Figure 2 provides an internal identifier that allows other systems to link to data entries in the information system. Each concept of the GPCR-OKB ontology that other systems could find useful to link to (e.g., Oligomer, Protein, Family, Ligand and IdentificationStudy in Figure 2) inherits the identifier attribute by extending the TrackedEntity concept. In Figure 2, this is shown graphically either with the open-ended arrow "is a" relation, or by the <<TrackedEntity>> special tag. The TrackedEntity concept can be extended with attributes to track accession codes over time and revision information.

#### 2. Molecules

In Figure 2, the concepts Oligomer, Ligand and Protein represent molecules. The Oligomer concept is defined by the following attributes: *oligomerName, protomers, studies, literatureReferences, rules, mechanismsOfActivation, isFormedInER, physiologicalRelevance*, and *structuralModels*. The attributes *oligomerName, protomers*, and *literatureReferences *have already been described in the previous section. Other attributes of the Oligomer concept are described below.

##### 2.1 Oligomer

The attribute *rules *stores information on whether the Oligomer conforms to the list of recommendations recently stipulated by the NC-IUPHAR subcommittee for the recognition and nomenclature of GPCR multimers (described in [13]). The concept NciupharRules, also shown in Figure 2, indicates which of the NC-IUPHAR rules the given Oligomer satisfies. These rules, which ensure the existence of an oligomer, are: 1) evidence of physical association; 2) identification of a specific oligomeric function; and 3) evidence for the existence of the oligomer *in vivo*. Each of the three rules has an attribute associated with it. Each attribute is of a boolean type, meaning that it can either be TRUE or FALSE.

Oligomers may be assembled either in the endoplasmic reticulum (ER) and then exported to the cell membrane, or the individual protomers that form the oligomer may be exported separately to the cell membrane and the oligomer be assembled there. The attribute *isFormedInER *refers to the formation of the oligomer in the ER. A prototypical example of an oligomer formed in the ER, and subsequently exported to the membrane (receptor maturation/ontogeny) is the heterodimeric γ-aminobutiric acid GABA_B _receptor. In fact, subunit GABA_B1 _is usually retained in the ER, and only its dimerization with subunit GABA_B2 _allows for the export of the functional heterodimer to the plasma cell membrane [33-35]. Similar behavior has recently been described for some members of the class A GPCRs such as αB and αD-adrenergic receptors heterodimers [36], vasopressin V1a, V2, and the oxytocin receptors homo- and heterodimers [2], C5a homodimers [37], and others. Thus, for these oligomers, the *isFormedInER *attribute is TRUE. When new techniques become available that allow the unambiguous verification of export to the cell membrane, then such data will also be included in the information system.

Other attributes of the Oligomer concept, such as *studies*, *mechanismsOfActivation *and *physiologicalRelevance*, describe more detailed information related to the oligomer; any structural information available for the oligomer is detailed in the attribute *structuralModels*. These information concepts are depicted in Figures 3 and 4 and will be discussed in the following sections.

##### 2.2 Ligand

In Figure 2, the Ligand concept refers to any molecule that binds to a receptor (e.g., small molecules or peptides). Ligands have a name meant for display in the user interface (*ligandName*), and a collection of external references (*databaseReferences*). These references make it possible for users to follow links to databases where the small molecule, peptide, or protein ligand is described in detail (e.g., PubChem [38]). We can also represent novel compounds by their canonical SMILES representation (*smilesStructure*). An introduction to canonical SMILES is provided by [39].

##### 2.3 Protein

The Protein concept represents individual protein chains or protomeric units, defined by the attribute *proteinName*. The *organism *attribute stores the Latin names for the source organism. A more detailed description of the Protein is stored in the *description *attribute. The *databaseReferences *attribute of protein makes it possible to non-ambiguously identify the protein in external databases. The *oligomers *attribute lists the Oligomers in which the protein participates. The attribute *tmTopology *defines the location of the transmembrane segments (TM) used when determining the generic numbering attributes (see below), and also supports creating snake-like plots of the oligomerization interface where interacting residues are highlighted [40]. Each TM is encoded by an instance of SecondaryStructureElement (see below). Protein is further qualified with the most specific GPCR *family *to which it belongs (see below). Note that we use the term 'family' for any set of related proteins on a number of different levels which include super-families, families and classes of proteins. This simplification makes it possible to re-use the same ontology concept (called 'Family') for all levels of hierarchy.

##### 2.4 SecondaryStructureElement

This concept stores information about each individual secondary structure element in a GPCR protein, such as helices. The helical boundaries for family A GPCRs are based on the alignment with the rhodopsin crystal structure. Helical boundaries for all other families of proteins will be defined by secondary structure predictions and hydropathy profiles. Its attributes include *startPosition *(the absolute position in the protein where the element starts), *endPosition *(the absolute numbering of the end of the element) and *elementName *(the conventional name given to the secondary structure element. For example, the third transmembrane segment of the bovine μ-opioid receptor would be stored as *startPosition *= 107, *endPosition *= 139 and *elementName *= TM3.

##### 2.5 Family

The Family concept supports a typical hierarchical organization of protein families (*parent *and *children *attributes). For instance, the opioid receptor family has three children (= opioid receptor type D, opioid receptor type K, and opioid receptor type M). The opioid receptor type D family then has one parent (= opioid) and one child (= δ-opioid receptor). The name of the family is stored in *familyName*, and the proteins that comprise the family are stored in *memberProteins*. Any references in online databases to this family are stored in *databaseReferences*.

#### 3. Studies of oligomer and interface identification

##### 3.1 IdentificationStudy

The IdentificationStudy concept describes the studies and methods that led to the identification of an oligomer or the interacting structural domains of the protomers at the oligomeric interface. The *methodType *attribute describes the type of method used in the study and is described in more detail in the following section. The *phenotype *attribute indicates if any phenotypic changes were observed in the referenced study. (The concept PhenotypicChange is described in detail in Figure 4 and is discussed below). The *literatureReferences *attribute lists one or more publications describing each particular study. When the cell type in which the identification has been carried out is known, it is represented by the *cellType *attribute, which is encoded by the Cell Ontology [41]. When available, the attributes *interfaceDiscovered *and *residuesFound *indicate the parts of the protomers that have been found to interact in the Oligomer in the given study (see below). A prototypical example of the attribute *interfaceDiscovered *is offered by rhodopsin dimers [42]. Specifically, based on the AFM-based atomic model of rhodopsin dimers (MethodType = AFM, *literatureReferences *= [43]) the intradimeric contacts have been described as involving TM4 and TM5 [42] (*interfaceDiscovered *= {TM4, TM5}, where TM4 and TM5 are stored as instances of SecondaryStructureElement in the *composedOf *attribute of InteractionDomain, described below). Of note, a recent crystal structure of a photoactivated deprotonated intermediate of bovine rhodopsin has shown TM1 as an alternative dimerization interface [44]. The IdentificationStudy concept also includes a *measurements *attribute, which records how various experimental measurements obtained in the study are affected by treatment with ligands (see LigandEffectOnMeasurement, below). This attribute stores the effect of ligands on the experimental measurements performed in the study (see below, and Figure 4).

##### 3.2 InteractionDomain

The InteractionDomain concept groups functionally or topologically related sets of residues that may or may not be adjacent in sequence. Other InteractionDomain concept attributes include *domainName *(e.g., extracellular, intracellular, transmembrane), and the *protomer *attribute that indicates what protein the defined interaction domain belongs to. If the domain can be mapped to defined secondary structure elements of the protomer, this information is stored in the *composedOf *attribute. For example, an InteractionDomain with *domainName *= extracellular would include four SecondaryStructureElements in the *composedOf *attribute, where the *elementName *would be N-term, EC1, EC2 and EC3, respectively. In the above example of rhodopsin dimers, there would be one InteractionDomain instance defined: with *protomer *= rhodopsin, *domainName *= TM4_5, and two instances of SecondaryStructureElement in the *composedOf *attribute, one with *elementName *= TM4, the other with *elementName *= TM5. The *interactingResidues *attribute is used in cases where it is known which residues in a domain interact at the interface (see below).

##### 3.3 Residue

Information on the residues that are involved in the interaction at the interface is limited. To accommodate cases where this information is known we have included a Residue concept. The Residue concept represents a single residue. Similarly to the InteractionDomain concept, the *protomer *attribute of the Residue concept indicates to which protein the residue belongs, since a residue at the dimerization interface may belong to any of the protomers of the Oligomer. The *protomer *attribute for the various residues of a particular interface would be the same protein for homodimers, and either A or B for heterodimers. The *position *attribute is the absolute position of the residue in the protomer sequence, *residueCode *is the one letter code for the residue, and *tmIndex *and *residueIndex *support the generic numbering system described in [21,22]. For instance, in the case of the dopamine D2 receptor, cysteine cross-linking studies have identified specific residues of TM4 at the interface of dopamine D2 receptor homodimers. One of these residues is Cys168^4.58 ^at the extracellular end of TM4. Thus, this information can be represented as follows in the ontology: MethodType = Cross-linking, *interfaceDiscovered *= InteractionDomain:*domainName *= TM4, *residuesFound *= Cys168^4.58^, *literatureReferences *[45]). In the Residue concept, residue Cys168^4.58 ^would be represented by *protomer *= dopamine D2, *residueCode = *C, *position *= 168, *tmIndex *= 4 and *residueIndex *= 58.

##### 3.4 MethodType

The MethodType concept has three attributes: *methodName*, *explanation *and *isComputational*. The attribute *methodName *stores the abbreviation or common name of the method; the *explanation *attribute stores the description of that method. The attribute *isComputational *refers to whether the method is computational or experimental. The concept MethodType is further subdivided into CreationMethod and IdentificationMethod (see Figure 3). IdentificationMethod stores any method used to identify the existence of an oligomer. CreationMethod stores any method that can guide the prediction of the oligomeric interface leading to a 3D model of the complex. For example, a subclass of the IdentificationMethod concept describing the experimental method coimmunoprecipitation would have *methodName *= COIP, *explanation *= coimmunoprecipitation and *isComputational *= FALSE. Another subclass to be considered may be FRET (also shown as an example in Figure 3). The CreationMethod concept includes, but is not limited to, experimental or computational techniques such as AFM, and CMA (see Figure 3). Methods such as cross-linking can be considered to be examples of both CreationMethod and IdentificationMethod.

##### 3.5 LigandEffectOnMeasurement

Treating an oligomer with a ligand can change the intensity of a measured signal (i.e., amount of co-immunoprecipitation, BRET signal, etc.). The concept LigandEffectOnMeasurement encodes this information for each ligand tested in a study (the type of measurement tested is specified in the IdentificationStudy instance that refers to this measurement). This concept includes three attributes: *ligandUnderTest*, *measuredEffect *and *phenotypicChange*. The attribute *ligandUnderTest *stores the ligand used for treatment. The attribute *measuredEffect *stores how the measured signal changes upon ligand treatment (INCREASED, DECREASED, UNCHANGED, NOT_TESTED). If there was also a phenotypic change recorded with this ligand (relative to any of the component protomers), this change would be stored in the *phenotypicChange *attribute (see Section 6 for details). For example, four instances of LigandEffectOnMeasurement would be used to encode the BRET measurements made on cholecystokinin (CCK) type A receptor in an experiment carried out by Cheng *et al*. [46], where the carboxyl-terminal was tagged with Renilla luciferase or yellow fluorescent protein. In the presence of the two agonist ligands CCK and gastrin-4 (= *ligandUnderTest*), the signal was observed to be reduced and the *measuredEffect *in both cases would be stored as DECREASED. In the presence of the partial agonist [CCK-26-32]-*O*-phenylethyl ester (*OPE*), the effect was also reduced (although to a lesser degree). This change would also be stored as *measuredEffect *= DECREASED. When the antagonist [(D-Trp30)CCK-26-32]-*O*-phenylethyl ester (*D-Trp-OPE*) was used, the *measuredEffect *was UNCHANGED [46].

While a number of published articles have interpreted ligand effects on such measurements as evidence of changes in oligomerization state, our ontology puts the emphasis on the storage of experimental results and leaves the interpretation of these results to the end-users of the information.

#### 4. Molecular structures and computational simulations

Figure 3 presents the concepts that encode structural information and the results of simulation studies on GPCR oligomers. The *structuralModels *attribute of Oligomer identifies any type of 3D structural information that relates to the Oligomer.

##### 4.1 MolecularStructure

Molecular models or experimentally determined structures are encoded as instances of the concept MolecularStructure. The *creation *attribute of MolecularStructure describes the protocol followed to create the model, or indicates the experimental technique used to determine the structure. Only instances of the CreationMethod subclass of the MethodType concept are used to store information about the methods used to determine a 3D structure. Examples of instances of the concept MolecularStructure are: a) the different crystal structures of the extracellular ligand-binding region of the homodimeric metabotropic glutamate receptor subtype 1 [47,48], b) the semi-empirical model of mouse rhodopsin oligomers deduced from AFM studies in native disk membranes [43], and c) correlated mutation-based models such as the ones described for opioid receptor [49] or dopamine D2 receptor [45] homodimers. There are two attributes to store references regarding the MolecularStructure. The attribute *databaseReferences *includes references to other databases with information about this structure while the *literatureReferences *stores any articles from the scientific literature about the structure. Although the two types of references are represented in the same way in our ontology, it is useful to distinguish the two types.

The *predictions *attribute stores structural or functional hypotheses that were derived from the analysis of the molecular structure. A given molecular structure can be used in multiple computational analyses, and these are stored in the *analyses *attribute. Similarly, several molecular simulation studies (e.g., molecular dynamics, normal mode analysis, etc.) can be performed on the same structure, and are also listed under the attribute *analyses*. Optionally, an *images *attribute of MolecularStructure (not included in the ontology) could offer a way to present users with illustrations of structural information.

##### 4.2 Analysis

The Analysis concept stores information about all computational procedures that have been used to create and to analyze the 3D molecular structure of the oligomer. It includes three attributes: *technique*, *programNamesUsed *and *literatureReferences*. The attribute *technique *stores the name of the technique used in the analysis, and *programNamesUsed *lists the names of the computational packages that were used in the analysis. Rather than detailing all the minutiae of the analysis in the ontology, the interested user will be directed to the journal articles (listed in *literatureReferences*) that describe the analysis.

#### 5. Mechanism of activation

The concept MechanismOfActivation in Figure 4 allows the storage of information about mechanistic hypotheses of oligomeric activation.

Current models of ligand binding and signal transduction by GPCR dimers can be described as symmetric or asymmetric depending on whether or not both protomers of the dimer undergo similar or different conformational changes upon activation. The ligand that is responsible for the activation mechanism being described in a given instance of MechanismOfActivation is stored in the *activatingLigands *attribute. The list of protomers that undergo a conformational change leading to activation of the oligomer is stored in the *activatedProtomers *attribute. The attribute *undergoesInterfaceRearrangement *indicates whether or not this conformational change is known to involve domains or residues at the oligomerization interface. A summary of the specific types of structural change within a protomeric unit will be contained in the attribute *descriptionOfChange *(e.g., "TM6 moves away from TM3"). Details will be found in the associated publication, which information is stored through the attribute *literatureReferences*. The attribute *deducedFromStudies *provides information about the computational/experimental studies that suggested the specific mechanistic hypothesis of activation.

The attribute *bindingSiteOccupancy *indicates how many ligand binding sites are occupied in a given GPCR oligomer. The attribute *isAsymmetric *allows discrimination between hypotheses of symmetric or asymmetric activation for a given GPCR oligomer. The particular case of asymmetric activation in which one protomer of a GPCR dimer is not capable of G-protein coupling, whereas the other protomer can bind the G-protein but not the ligand will be denoted by the attribute *isTransactivated *set to TRUE. On the other hand, if the ligand-bound protomer that undergoes a conformational change is also the one that activates the G-protein, the mechanism of activation is referred to as cis-activation, and the attribute *isCisactivated *will be set to TRUE. Both, *cis- *and *trans-*activation attributes are not mutually exclusive and could both be TRUE. Finally, the stoichiometry of the signaling complex, i.e., how many GPCRs and G-proteins form a functional GPCR oligomer, is expressed by use of the attributes *numberGPCRs *and *numberGProteins*.

The most unambiguous example of *trans*-activation has been described for the GABA_B1_-GABA_B2 _heterodimer, where the ligand-bound subunit (GABA_B1_) does not appear to couple to the G-protein, but the other subunit (GABA_B2_) within the hetero-dimer can bind the G-protein, but not the ligand [50]. Thus, for the GABA_B1_-GABA_B2 _heterodimer the attribute *isTransactivated *would be set to TRUE. On the other hand, studies of the histamine H1 receptor and α1B-adrenoreceptor homodimers [51] provide mechanistic hypotheses of *cis-*activation (i.e., the protomer that binds the ligand and undergoes the conformational change is the same protomer that activates the G-protein). Hence they will be identified by setting *isCisactivated *= TRUE. Analysis of GABA_B _chimeric subunits seems to suggest that in other homodimeric classes of GPCRs, in which both "subunits" are capable of binding ligand and signaling to G-protein, both *cis*- and *trans-*activation may take place [50]. In fact, in the case of the luteinizing hormone receptor dimer the binding of hormone seems to activate adenylyl cyclase through the same receptor TM bundle (*cis-*activation), as well as *trans-*activate through the TM bundle of an adjacent receptor [52]. Therefore in this example *isCisactivated *= TRUE and *isTransactivated *= TRUE.

A more generic example of asymmetric activation (*isAsymmetric *= TRUE) is represented by the metabotropic glutamate (mGlu) homodimers [53,54]. Using a new class of synthetic allosteric modulators (information about them would be contained in the LigandEffectOnMeasurement concept, see above), recent studies have suggested that only one heptahelical domain of the mGlu homodimer can undergo activation. In this case of asymmetric activation, it has also been demonstrated that the binding pockets of both ectodomains of the mGlu homodimer must be occupied for full activation of the system (*bindingSiteOccupancy *= 2) [55].

An elegant experimental study of the leukotriene B4 receptor (BLT1) suggests that a functional GPCR complex is composed of one heterotrimeric G-protein and one GPCR dimer [56] (*numberGPCR *= 2; *numberGProtein *= 1). Indeed, one protomer only is active in such dimers in the presence of the G-protein, and this is sufficient for full G-protein activation (see Damian *et al*. [57]). Stoichiometry may become even more complicated in the case of GPCR oligomers, as recently suggested by Palczewski's lab based on inferences from AFM studies [58]. As per the specific conformational changes that BLT1 undergoes, experimental evidence suggests a rearrangement of TM6 [56]. This information will be stored under *descriptionOfChange = *"TM6 rearrangement".

Examples of *undergoesInterfaceRearrangement *= TRUE have been reported for the mGluR1 and dopamine D2 receptors. Specifically, crystallographic structures of glutamate-bound and unbound forms of mGluR1 [47,48] provide evidence for the rearrangement of the extracellular dimeric conformations, which may subsequently induce a conformational change in the cytoplasmic regions [59]. Finally, cross-linking studies (= *deducedFromStudies*) of dopamine D2 receptor in the presence of agonists and inverse agonists also suggest a conformational rearrangement of the TM4 homodimeric interface upon activation [60].

#### 6. Phenotypic changes

The *phenotype *attribute of IdentificationStudy encodes the change of phenotype associated with oligomerization (the association observed in the study described by IdentificationStudy). Our ontology only encodes *changes *in phenotype that are observed in the presence of different combinations of GPCRs with their associated ligands [2]. A change occurs if the phenotype of the Oligomer is significantly different from the phenotype of either one of the protomers (or the homodimers of those protomers) that participate in the Oligomer. The *comparedWith *attribute stores the protomer relative to which the phenotypic changes stored in PhenotypicChange are being compared. Figure 4 shows that the GPCR oligomerization ontology distinguishes between three types of phenotypic changes: internalization, ligand binding, and signaling changes. The PhenotypicChange concept does not include a *literatureReferences *attribute since they are already listed in the IdentificationStudy concept.

##### 6.1 Internalization

The ontology tracks reported changes to internalization of the oligomer with respect to internalization of the monomer using four attributes: *ligandInvolved*, *readoutProtomer*, *boundProtomer *and *isInternalized*. Internalization, trafficking from the cell membrane, is usually observed upon ligand stimulation, and different effects are often observed with different ligands.

Each tested ligand (attribute *ligandInvolved*) is associated with its bound protomer (attribute *boundProtomer*), and to the internalization effect observed (*isInternalized*) on the protomer being studied (*readoutProtomer*).

An example of an instance of the Internalization concept is provided by confocal microscopy studies carried out on co-expressed sst2A somatostatin receptor and μ-opioid receptors. Treatment with a sst2A-specific ligand, L-779,976, induces the internalization of both the sst2A and μ-opioid receptors, but treatment with a μ-opioid receptor specific ligand, [D-Ala2, N-Me-Phe4, Gly5-ol]-enkephalin (DAMGO), only induced the internalization of μ-opioid, not of sst2A. These three results would be stored as 1) *boundProtomer *= sst2A, *ligandInvolved *= L779,976, *readoutProtomer *= {sst2A, μ-opioid} and *isInternalized *= TRUE; 2) *boundProtomer *= sst2A, *ligandInvolved *= DAMGO, *readoutProtomer *= sst2A and *isInternalized *= FALSE; 3) *boundProtomer *= μ-opioid, *ligandInvolved *= DAMGO, *readoutProtomer *= μ-opioid and *isInternalized *= TRUE [61].

##### 6.2 Ligand binding

The concept LigandBinding encodes differences in the binding of a given ligand to the Oligomer with respect to the protomer. This concept is defined by the following attributes: a) the ligand tested in the pharmacological assay (*ligandUnderTest*), b) the property of ligand action, indicating whether the ligand is an agonist, partial agonist, antagonist, neutral antagonist or inverse agonist (*ligandProperty*), c) the protomer (or protomers) that the ligand is observed to bind (*protomersBound*), d) the measured change in ligand affinity to the oligomer compared to the protomer (*affinityChange*), and e) the presence of demonstrated ligand binding crosstalk (*bindingXTalk*) – see section 6.4. The helper concept MeasuredVariation records if the affinity was: INCREASED, DECREASED, NOT CHANGED, or NOT_TESTED.

Results of μ- and δ-opioid receptor co-expression can be used as an example of the type of information that attributes of the LigandBinding concept will contain. Experimental data indicate that the μ-δ opioid oligomeric complex exhibits a 10-fold lower affinity for both the μ-selective agonist DAMGO and the δ-selective agonist [D-Pen2, D-Pen5]enkephalin (DPDPE) (*comparedWith = *μ-opioid receptor and δ-opioid receptor, respectively) [62]. The information can be represented by two instances of LigandBinding as follows: 1) *ligandUnderTest *= DAMGO, *protomersBound *= {μ-opioid receptor}, *affinityChange *= DECREASED; 2) *ligandUnderTest *= DPDPE, *protomersBound *= {δ-opioid receptor}, *affinityChange *= DECREASED. On the other hand, endogenous opioid peptides such as endomorphin-1 (= *ligandUnderTest*; *protomersBound *= {μ-opioid}) and Leu-enkephalin (*protomersBound *= {δ-opioid, μ-opioid}) have enhanced affinity (*affinityChange *= INCREASED) for the heteromeric opioid receptor complex (*comparedWith = *μ-opioid receptor). [62].

##### 6.3 Signaling

GPCRs mostly signal through heterotrimeric G proteins although increasing evidence suggests that GPCRs may also function in a G-protein-independent manner. In this section we only refer to classical G protein-dependent signaling pathways, although the ontology can incorporate information about G protein-independent signaling pathways as well.

GPCR oligomers can signal through different pathways than a GPCR monomer. The Signaling concept makes it possible to encode such differences in downstream signal transduction. Encoding of this concept assumes that a molecule (in attribute *molecule*) that belongs to a pathway (in attribute *pathway*) downstream of the receptor has been tested (the type of assay can be described in the attribute *measurementType*). The attribute *changeInMeasurement *records either how the output of the assay differs from the monomer to the oligomer or the effect that different cross-talk ligands have on specific ligand-mediated signaling processes (see *signalingXtalk *attribute below). For instance, if the levels of cAMP are tested, and the corresponding assay reports that cAMP levels are higher for the oligomer than for the monomer, then *changeInMeasurement *will have the value INCREASED. The attribute *activatingLigand *records which ligand was used to activate the receptor. The attribute *isDesensitized *is used to report the loss of responsiveness of the protomer to the continuing or increasing dose of *activatingLigand*. The attribute *isPhosphorylated *contains information about whether the GPCR receptor is phosphorylated or not. Further, the attribute *signalingXTalk *indicates if the signal transduction change corresponds to crosstalk between signaling pathways mediated by the protomers of the Oligomer (see section 6.4). Finally, changes in G-protein specificity are accounted by the attribute *gProteinType*. This attribute is part of the Signaling concept rather than Oligomer as the G-protein specificity is dependent on the ligand.

For example, in cells co-expressing sst2A and μ-opioid, addition of L779,976 to the system is able to promote phosphorylation and desensitization of both receptors, compared with the μ-opioid receptors expressed alone. This is stored as *comparedWith *= μ-opioid, *activatingLigand *= L779,976, *readoutProtomer *= {sst2A, μ-opioid}, *isPhosphorylated *= TRUE, *isDesensitized *= TRUE; 2) [61].

An example of possible changes in G-protein specificity can be illustrated by the effect of agonist stimulation on co-expressed dopamine D1 and D2 dopamine receptors. Stimulation results in an increase of intracellular calcium levels via a signaling pathway not activated by either receptor alone or when either one of the co-expressed receptors was activated by a selective agonist. Furthermore, calcium signaling by D1-D2 dopamine receptor co-activation was abolished following treatment with a phospholipase C inhibitor but not with pertussis toxin or inhibitors of protein kinase A or protein kinase C, indicating coupling to the Gα_q _pathway. This information would be stored as *molecule *= Ca^2+^, *pathway *= phospholipase C dependent pathway, *readoutProtomer *= {dopamine D1, dopamine D2}, *gProteinType *= Gα_q _[63].

The use of some of the other attributes in Signaling can be demonstrated using the experiments by Breit *et al*. on heterologous systems co-expressing sensory neuron-specific receptors subtype 4 (SNSR-4) and δ-opioid receptors [64]. When these receptors are co-expressed in HEK-293 cells, each receptor acts as an independent unit. In the heterologous system, the SNSR4 selective bovine adrenal medulla peptide BAM22 produces similar amounts of phospholipase C activation as it does in cells expressing only SNSR4. This is stored as: *comparedWith *= SNSR4, *molecule *= phosphoinositol, *pathway *= phospholipase C, *measurementType *= induced accumulation of total phosphoinositols, *activatingLigand *= BAM22, *gProteinType *= Gα_q_, *changeInMeasurement *= UNCHANGED.

##### 6.4 CrossTalk

The attributes *signalingXTalk *and *bindingXTalk *are used to describe changes in the affinity or signaling of the *ligandA *(i.e., the ligand bound to protomer A of the oligomer, which should be the same as the value of the attribute *ligandUnderTest *of the referring instance of LigandBinding, or *ligand *in Signaling) in the presence of another ligand (i.e., a ligand bound to protomer B of the oligomer). The concept CrossTalk includes attributes that account for the specific ligands (*ligandA *and *ligandB*) and protomers (*protomerA *and *protomerB*) involved in changes in affinity (when referred to LigandBinding or signaling when talking of signalingXtalk) due to crosstalk (*changeInProtomerA*).

An example of bindingXTalk (sometimes called positive/negative cooperativity) can be seen in the treatment of cells or native membranes containing both μ-opioid (= *protomerA*) and δ-opioid (= *protomerB*) receptors [65,66] with low doses of a selective δ-opioid ligand such as TIPPΨ (H-Tyr-TicΨ [CH_2_NH]Phe-Phe-OH) (=*ligandB*) produces a two-fold increase in binding affinity (*changeInProtomerA *= INCREASED) of the μ-opioid agonist DAMGO (= *ligandA*). Figure 5 illustrates pictorially this example together with three other experiments carried out in the same cell system.

Similarly, for signalingXTalk, *in vivo *analgesia studies have shown that pretreatment of mice with the δ-selective agonist naltrindole NTI lowers the antinociceptive potency values (ED50) of compounds such as MDAN-19 and MDAN-16 to either δ – or μ-opioid receptors, but has no effect on MDAN-21 potency [67]. This MDAN (μ-agonist-δ-antagonist) series of bivalent ligands has been designed by combining pharmacophores from oxymorphone (μ-opioid receptor agonist) and NTI (δ-opioid receptor antagonist). Thus, this information will be entered as follows: *ligandA *= MDAN21, *protomerA *= μ-receptor, *ligand B *= NTI, *protomerB *= δ-receptor, *changeInProtomerA *= UNCHANGED, or MDAN16, *protomerA *= μ-receptor, *ligand B *= NTI, *protomerB *= δ-receptor, *changeInProtomerA *= DECREASED.

#### 7. Physiological relevance

A growing body of evidence refers to the existence of GPCR oligomers in native tissue. This information is extremely important as it relates to the physiological relevance of the GPCR oligomer under study. Whether or not all protomers of an oligomer are identified in the same cell or within the same subcellular compartments (*colocalizationEnvironment*) will be indicated by the *isColocalized *attribute. Specific functional properties for the GPCR oligomers identified in native tissue (e.g., positive or negative allosteric interaction between the two binding sites of a GPCR dimer, information about a ligand specific to the dimer, activation of a specific signaling pathway, or specific internalization or desensitization properties) will be stored via the *inVivoPhenotypicChange *attribute. As the use of knock out animals or RNAi technology may also provide key information on the existence of GPCR oligomers *in vivo*, this information will be stored as well using the *geneKnockoutResponse *attribute.

The case of GABA_B _receptors is used here to provide an example of the physiological information that can be stored using the attributes described above. For instance, to indicate that subunits GABA_B1 _and GABA_B2 _are co-localized in the brain [34], we will use the attributes *isColocalized *= TRUE and *colocalizationEnvironment = *brain. The deletion of either GABA_B1 _[68] or GABA_B2 _[69] in mice leads to similar phenotypes with no detectable GABA_B _responses (=*geneKnockoutResponse*).

## Discussion

As data about GPCR oligomerization accumulate in the scientific literature, it becomes appropriate to create an electronic repository to facilitate the browsing, searching and integration of relevant data. However, the construction of such a resource is not trivial because the resource should offer information about many various aspects of GPCR oligomerization and how these aspects interrelate. To enable the construction of a GPCR oligomerization information system that is as complete and responsive to the needs of the GPCR community as possible, we have gathered the requirements of such a system from experimental and computational experts in the GPCR field. Based on an interdisciplinary dialog, we have created an ontology that formalizes information concepts and concept relationships required to store the different aspects of GPCR oligomerization in an electronic repository. These concepts are formally defined in the OWL language, a W3C standard for ontology exchange [70] recognized by the Open Biomedical Ontologies consortium (OBO) [71]. Since XML-based exchange formats can be generated from an ontology (e.g., as is done in the BioPax project [72]), the GPCR Oligomerization ontology can also support the exchange of data among other GPCR oligomerization-aware systems.

Many ontologies proposed under the OBO consortium can be reused in a variety of applications (e.g., the Cell Ontology provides an ontology of cell types useful for a variety of bioinformatics resources [41] and is re-used in the ontology described here, as part of the IdentificationStudy concept). However, many successful ontologies have a more restricted focus, such as the EcoCyc ontology. The EcoCyc ontology offers a consensual view of how information about the *E. coli* metabolic pathways could be stored electronically [73,74]. The GPCR Oligomerization ontology is similarly necessarily restricted in its focus, having been designed to make possible the development of a GPCR oligomerization information system.

Although reusability of the GPCR Oligomerization ontology is likely to be limited, we expect that ideas expressed in this ontology, for instance about how phenotypic changes are represented (see below and Figure 4), may be useful in other fields. Although there are other large-scale projects underway to define phenotype ontologies (i.e., the mammalian phenotype ontology (MPO) [75]), the phenotypic representation required by the GPCR oligomerization system falls outside of the utility of the MPO ontology. Specifically, (1) the current focus of MPO is on phenotype observed at the organ, organism and cell levels. These phenotypes do not match the pharmacological and signaling phenotypes most often considered in the GPCR field; (2) MPO models phenotypes, while reports available in the literature for GPCR oligomers generally characterize phenotypic changes (e.g., the affinity of ligand X is increased in the oligomer compared to that in the protomer alone); (3) the tree structure of the MPO assumes that each phenotype is represented as a distinct concept of the ontology. Categorizing each distinct phenotype with a different concept of the ontology in this way does not support computational comparisons of phenotypic changes required by a GPCR oligomerization information system. For example, obtaining the list of ligands whose binding to the Oligomer is increased when compared to binding of the same ligand to the protomer is possible with our ontology, but not with the relations offered by MPO.

In contrast to the MPO hierarchy of phenotypes, the solution that we retained for the GPCR Oligomerization ontology models phenotypic changes as concepts which are endowed with attributes that support explicit comparison and aggregation of phenotypic changes (a phenotypic change that follows this approach is illustrated in Figure 5). In this respect, the choices made in the GPCR Oligomerization ontology are driven by the types of computation that we expect an information system will perform with GPCR oligomerization information. Since protein oligomerization and its impact on downstream signaling pathways is a regulation and signaling paradigm that occurs in a variety of non-GPCR systems, we anticipate that our representation of phenotypic changes will be reusable in other fields, e.g., protein kinases. The phosphorylation of a protein by a protein kinase may change its enzymatic activity, cellular location or association with other proteins. The GPCR Oligomerization ontology models such changes at an intermediate level of detail which omits mechanistic and precise stoichiometric details, yet captures the information most useful to scientists who study these regulation mechanisms experimentally.

Although the ontology presented here will continue to develop as studies of GPCR oligomerization progress, the version of the GPCR Oligomerization ontology presented in this manuscript is expected to facilitate the development of future GPCR oligomerization information systems. The information that is entered into systems based on this ontology can be manually extracted from the literature by expert curators. Because of the possibility of several different interpretations of the primary data presented in an article, our ontology focuses on primary experimental data that are objective and not subject to interpretation. In addition to primary data, reported oligomeric complexes of GPCRs that satisfy one or more of the rules recently specified by NC-IUPHAR will be properly highlighted.

It is worth underscoring that attributes of inference have been avoided in the construction of this ontology, in order to offer as objective and neutral information as possible. The user will find always a referenced article or articles for further reading and discussion on the topic of interest. Our main goal has been to reflect the information as close to the data as possible so the user can compare related studies and make his or her own inferences or judgments on the conclusions provided by the study or studies available. For example, in Angers *et al*., isoproterenol was suggested to induce homodimerization of the β2-adrenergic receptor based on inferences from BRET results [76]. However, further studies with a modified and newer version of BRET reported that stimulation with the same agonist did not promote consistent change in the BRET saturation curves. Thus, the authors concluded that the dimers form constitutively, and that receptor activation does not affect their oligomerization state [77]. In this ontology both examples will be collected as part of identification studies related to β2-adrenergic receptor homodimers. However, only the increased or unchanged BRET signal in the presence of that agonist will be reported, with omission of any inference about oligomer formation.

The construction of the GPCR Oligomerization ontology may also lend itself to the possibility of automatically extracting information that can be directly added to a database based on that ontology. Curated databases (such as the one under development in our laboratories) require curators to read and interpret a manuscript to extract information that will be added to the database. A more collaborative strategy could be proposed where structured information would be embedded in articles in such a way that the extraction of information can be automated and will not require human interpretation. The advantage of this strategy is that the structured information would be reviewed at the same time as the manuscript and that it would solve the problem of information extraction from the literature. The ontology described in this manuscript would support such a strategy because it is possible to automatically generate a structured language from an ontology that could be used to encode information for embedding in a manuscript. We note that while this strategy is possible, there are still a number of sociological barriers to its widespread use. In the meantime, the GPCR-OKB ontology presented here will be useful to database designers and curators who need to manage information about GPCR oligomerization.

## Conclusion

We have presented the requirements and ontology of an information system designed to manage the elements of information necessary to describe the phenomena of both homo- and hetero-oligomerization of GPCRs in a data-driven manner. The elements of information supported by the GPCR Oligomerization ontology include: i) links to other databases with information about GPCR protomers, ii) information about the experimental details of methods used to study GPCR oligomers, iii) experimental evidence for and computational predictions of the specific residues at oligomeric interfaces, iv) structural models of oligomers deduced from experiments or computer simulation studies; v) information about potential mechanisms of activation of GPCR oligomers; vi) information about functional roles of GPCR oligomers and the various phenotypical changes that occur compared to the individual subunits; vii) novel compounds that are proposed to selectively target GPCR oligomers; viii) the possible physiological relevance of GPCR oligomers.

The requirements and ontology described in this manuscript will facilitate the development of future GPCR oligomerization information systems that will integrate data generated by experimental and computational studies of GPCR oligomerization. The one under current development in our laboratories (GPCR-OKB) will be continually curated and maintained.

## Availability and requirements

The ontology for GPCR-OKB is available at .

## Authors' contributions

LS participated in the conceptual design of the ontology, created corresponding diagrams, and wrote the manuscript. MM helped to choose examples from the literature to illustrate the different attributes of the ontology, and participated in ontology design and manuscript drafting. MB, LD, SRG, MJL, GM, RN, KP, MP, JPP, and GV contributed to the manuscript draft with constructive criticism and helpful comments/suggestions. JAJ participated in requirement analysis, and helped refine the ontology design. FC and MF conceived the study, coordinated its design, and helped to draft the manuscript. All authors read and approved final manuscript.
